# Innovative Strategies in Hernia Mesh Design: Materials, Mechanics, and Modeling

**DOI:** 10.3390/ma18153509

**Published:** 2025-07-26

**Authors:** Evangelia Antoniadi, Nuno Miguel Ferreira, Maria Francisca Vaz, Marco Parente, Maria Pia Ferraz, Elisabete Silva

**Affiliations:** 1 Faculty of Engineering, University of Porto, 4200-465 Porto, Portugal; up202408581@edu.fe.up.pt (E.A.); nmferreira@inegi.up.pt (N.M.F.); mparente@fe.up.pt (M.P.); mpferraz@fe.up.pt (M.P.F.); 2 Associate Laboratory of Energy, Transports and Aerospace (LAETA), 4200-465 Porto, Portugal; fvaz@inegi.up.pt; 3 Institute of Science and Innovation in Mechanical and Industrial Engineering (INEGI), 4200-465 Porto, Portugal; 4 Ii3S—Instituto de Investigação e Inovação em Saúde, Universidade do Porto, 4200-135 Porto, Portugal

**Keywords:** hernia repair, postoperative complications, surgical mesh implant, biodegradable materials, drug-eluting mesh, finite element analysis

## Abstract

Hernia is a physiological condition that significantly impacts patients’ quality of life. Surgical treatment for hernias often involves the use of specialized meshes to support the abdominal wall. While this method is highly effective, it frequently leads to complications such as pain, infections, inflammation, adhesions, and even the need for revision surgeries. According to the Food and Drug Administration (FDA), hernia recurrence rates can reach up to 11%, surgical site infections occur in up to 21% of cases, and chronic pain incidence ranges from 0.3% to 68%. These statistics highlight the urgent need to improve mesh technologies to minimize such complications. The design and material composition of meshes are critical in reducing postoperative complications. Moreover, integrating drug-eluting properties into the meshes could address issues like infections and inflammation by enabling localized delivery of antibiotics and anti-inflammatory agents. Mesh design is equally important, with innovative structures like auxetic designs offering enhanced mechanical properties, flexibility, and tissue integration. These advanced designs can distribute stress more evenly, reduce fatigue, and improve performance in areas subjected to high pressures, such as during intense coughing, sneezing, or heavy lifting. Technological advancements, such as 3D printing, enable the precise fabrication of meshes with tailored designs and properties, providing new opportunities for innovation. By addressing these challenges, the development of next-generation mesh implants has the potential to reduce complications, improve patient outcomes, and significantly enhance quality of life for individuals undergoing hernia repair.

## 1. Introduction

**Figure 1:**
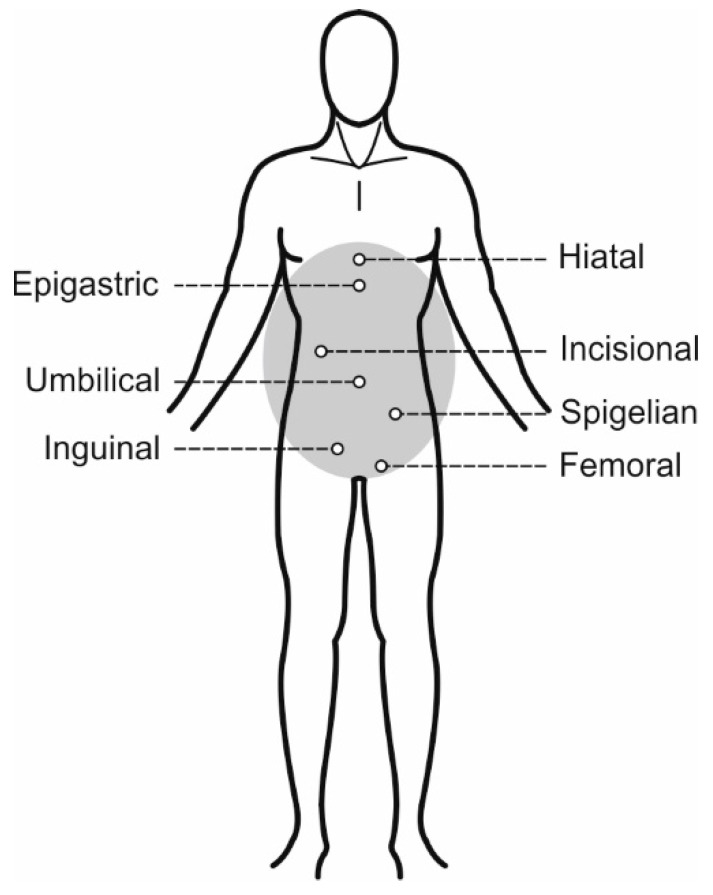
Types of hernia according to their location on the human body.

A hernia is a physiological problem where there is an organ prolapse through the wall of the cavity that is normally contained due to a weakness or opening, mainly of the abdominal wall [1]. The types of hernia vary, mostly according to their location on the human body (Figure 1). In the case of hernia, usually, a visible bulge on the skin is made, and it can appear in the inguinal cavity, umbilicus, femoral canal, and epigastrium, with the most frequent one being the inguinal with rates of 70–75% [1,2]. Following is the femoral with a range of 6–17%, the umbilical with 3–8.5%, incisional with 6.2% and other types, such as epigastric or hiatal, with a percentage of up to 8.6%.

The anatomical location of a hernia significantly influences the mechanical demands and clinical considerations for selecting an implant. Inguinal hernias are subject to relatively low mechanical stress and often benefit from lightweight polypropylene meshes to reduce pain and foreign body sensation. In contrast, ventral or incisional hernias typically require medium-weight mesh implants with sufficient strength, and, when placed intra-abdominally, a barrier to prevent bowel adhesions due to the direct contact with visceral organs [3].

Before the widespread adoption of synthetic mesh implants, hernia treatment relied on suture-based techniques, which were prone to complications and high recurrence rates. Efforts to reinforce these methods with metal or early plastics provided limited improvement and often led to infections and inflammations. These challenges highlighted the need for a more reliable, tension-free approach for hernia treatment, leading to the development of prosthetic mesh implants [4].

The introduction of synthetic mesh implants in hernia repair was made during the 1950s by Dr. Francis Usher, with a polypropylene (PP) mesh, which provided strong reinforcement to the abdominal wall, reducing the rates of hernia reappearance [5]. Before using meshes, the repair relied on suturing techniques, leading to very high recurrence rates. The mesh breakthrough was supported by both clinical trials and studies during the following decades, establishing the synthetic mesh as a standard in hernia repair surgery [5].

**Figure 2:**
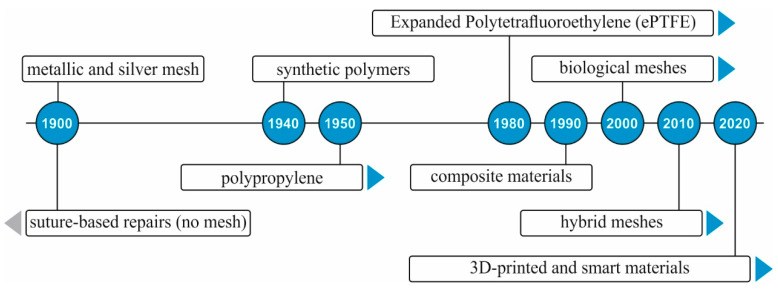
Timeline of the materials used on meshes for hernia repair.

Hernia mesh implants are categorized according to their material and the reactions they have with the body. Regarding the material, there are synthetic, biological, and hybrid meshes, as shown historically in Figure 2. Regarding pore size, mesh implants can be classified as macroporous, microporous, or composite. In terms of weight, they are categorized as heavyweight or lightweight. Additionally, based on their absorbability, meshes are categorized as absorbable, non-absorbable (permanent), or partially absorbable.

## 2. Complications

**Figure 3:**
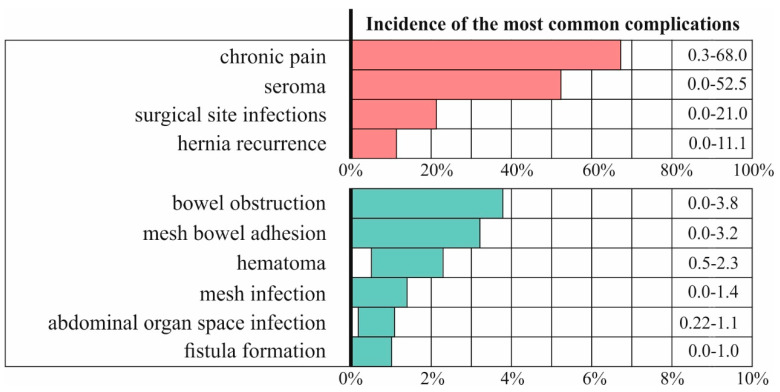
Complications of hernia repair after mesh implementation, according to the FDA [6].

Hernia mesh implant has been a revolutionary solution to hernia repair, but it remains associated with multiple complications, such as infections, adhesions, inflammations, chronic pain, and recurrence. According to the Food and Drug Administration (FDA), hernia recurrence rates can reach up to 11%, surgical site infections occur in up to 21% of cases, and chronic pain incidence ranges from 0.3% to 68% [6], as shown in Figure 3. Variations in the mechanical properties of mesh implants are associated with differences in the type and incidence of complications. The choice of material plays a critical role in the implant’s postoperative performance. Polypropylene (PP) is widely used due to its mechanical strength; however, it is prone to shrinkage and foreign body reactions [7]. In contrast, Polytetrafluoroethylene (PTFE) is associated with the highest reported infection rates, reaching up to 75% [7]. PTFE mesh implants tend to result in more bacterial infections due to their small pores and encapsulation rather than tissue incorporation.

Additionally, regarding the filament type, Robinson et al. [7] showed that multifilament meshes, though flexible, are more prone to bacterial colonization. A meta-analysis identified long operative time, smoking, emergency surgery, and a high American Society of Anesthesiologists (ASA) score as significant risk factors for mesh infections. Among these infections, up to 8% of cases involved common pathogens such as *Staphylococcus* spp. and *Enterococcus* spp. [8,9]. To reduce these risks, advancements in mesh technology, such as hybrid materials and drug-eluting coatings, have been under development [8].

Furthermore, surgical technique and the surgeon’s experience are critical factors influencing complication rates, with lower-volume surgeons showing higher incidences of intraoperative and postoperative adverse outcomes [10]. Specific surgical techniques, such as the Keyhole and Sugarbaker methods, used in parastomal hernia repairs, also influence outcomes, with prosthesis infections requiring mesh removal in a notable percentage of cases [11]. Additionally, patients with Crohn’s disease, especially those with fistulizing disease, have a higher risk of mesh-related complications [12].

## 3. Commercial Synthetic Hernia Mesh Implants: Mesh Geometries

Commercial mesh implants for hernia repair vary in material composition, pore size, weight, and elasticity, affecting their mechanical properties and biocompatibility. Examples of the commercially available meshes are presented in Table 1. The mesh implant geometry alters tissue integration, flexibility, and infection resistance. Meshes with larger pores present better fibrovascular ingrowth, while lightweight designs are shown to reduce foreign body reactions and post-surgical discomfort [13]. A feasibility study conducted by Panaro et al. presented a newly designed 3D mesh for inguinal hernia repair, featuring a dynamic, self-adjusting structure that improves anatomical adaptation, helping the body heal more effectively, without the need for fixation, as its 3D shape allows it to stay in place naturally [14]. The study used different types of Surgimesh™ implants with varying materials and properties. Experimental results in porcine and human cadaver models demonstrated good mesh tolerance, stable positioning, minimal inflammatory response, and effective integration with surrounding tissues [14].

Additionally, in a comparison of the geometry and properties of commercial meshes, such as Bard Mesh, Parietene, PROLENE Mesh, VYPRO Mesh, and DynaMesh-PP, it suggested that mesh geometry should be designed to mimic the natural mechanical behavior of the abdominal wall, as the anisotropic mechanical properties of both the abdominal wall and biomaterials were highlighted to manage the recurrence rates [15]. Another study examining commercial meshes—including Surgipro, Optilene, Infinit Mesh, Bard Mesh, Prolene, Parietene, Trelex, Prolite, Mersilene, Goretex, DynaMesh, and Vypro—discussed the physical structure and mechanical properties of knitted hernia mesh materials. The study emphasized that textile warp-knitted synthetic meshes significantly reduce hernia recurrence rates due to their flexibility, porosity, and mechanical compatibility with the abdominal wall [16]. Additional commercial mesh implants tested include Neomesh Soft^®^ (NM), Neopore^®^ (NP), Neomesh SuperSoft^®^ (SS), and Surgipro™ (SUR), where NP and NM meshes provide the best balance of flexibility and strength.

**Table 1:** Categories of commercially available mesh implants.

Synthetic Mesh Implants						
Mesh Name	Material	Filament Type	Pore Size	Weight (g/m^2^)	Manufacturer	References
Marlex	PP	monofilament	0.6 mm	95	Becton, Dickinson and Company (BD), Franklin Lakes, NJ, USA	[13,16]
Prolene	PP	dual-filament	1.0–2 mm	105	Ethicon, Johnson & Johnson, Somerville, NJ, USA	[13,16]
Trelex	PP	monofilament	0.6 mm	95	Meadox Medicals, Boston Scientific, Marlborough, MA, USA	[13,16]
Surgipro	PP	multifilament	0.9 mm	87	USSC, Norwalk, CT, USA	[13,17]
Lars	POL	multifilament	N/A	N/A	Corin Group, Gloucestershire, UK	[13]
Fluoropassiv	POL	multifilament	N/A	N/A	PFM Medical, Cologne, Germany	[13]
Bard Teflon	PTFE	multifilament	N/A	N/A	Becton, Dickinson and Company (BD), Franklin Lakes, NJ, USA	[13]
Goretex	ePTFE	N/A	0–25 μm	200–400	Gore Medical, Flagstaff, AZ, USA	[16]
Optilene	PP	monofilament	1 mm	36	B-Braun, Melsungen, Germany	[17]
Bard Mesh	PP	monofilament	0.0007–0.6500 mm^2^	97	Becton, Dickinson and Company (BD), Franklin Lakes, NJ, USA	[16]
Parietene LW	PP	monofilament	1.8 × 1.5 mm	38	Medtronic, Minneapolis, MN, USA	[16]
Prolite	PP	monofilament	0.8 mm	85	Atrium Medical Corporation, Hudson, NH, USA	[16,18]
Infinit Mesh	PTFE	monofilament	4.05 ± 0.22 mm^2^	70	N/A	[16]
Mersilene	POL	multifilament	0.6–1 mm	40	Ethicon, Johnson & Johnson, Somerville, NJ, USA	[16]
**Composite Mesh Implants**						
Vypro II	PP/Polyglactin 910	multifilament	3.0 mm	50	Ethicon, Johnson & Johnson, Somerville, NJ, USA	[17]
Dual Mesh	ePTFE	N/A	N/A	N/A	Gore Medical, Flagstaff, AZ, USA	[13]
Parietex	POL-Collagen	monofilament	2.4 mm	78	Covidien, Mansfield, MA, USA	[17]
Composix	PP-ePTFE	monofilament	N/A	N/A	Becton, Dickinson and Company (BD), Franklin Lakes, NJ, USA	[13]
Proceed	PP-Celluose	monofilament	N/A	N/A	Ethicon, Johnson & Johnson, Somerville, NJ, USA	[13]
Dynamesh	PP-PVDF	N/A	N/A	N/A	FEG, Aachen, Germany	[13]
Sepramesh	PP-Sodium	monofilament	N/A	N/A	Becton, Dickinson and Company (BD), Franklin Lakes, NJ, USA	[13]
Ultrapro	PP-Polyglecaprone	monofilament	3.5 mm	54	Ethicon, Johnson & Johnson, Somerville, NJ, USA	[17]
Ti-mesh	PP-Titanium	monofilament	N/A	N/A	PFM Medical, Cologne, Germany	[13]
C-Qur	PP-Ω3	N/A	N/A	N/A	Atrium Medical Corporation, Hudson, NH, USA	[13]

N/A: Not Available.

**Figure 4:**
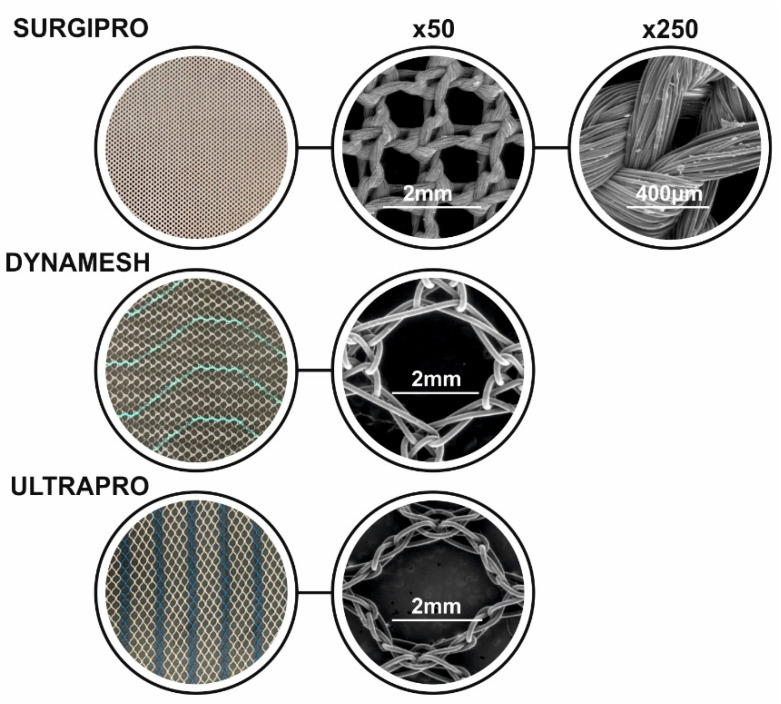
Macroscopic and microscopic views of three commercial hernia meshes (Surgipro, Dynamesh, and Ultrapro). Center and right panels show SEM images at ×50 and ×250 magnifications, respectively. Scale bars are included to illustrate differences in pore size, fiber arrangement, and surface morphology.

To complement the comparative data shown in Table 1, Figure 4 presents macroscopic and microscopic images of representative commercial meshes, highlighting their differences in pore structure and fiber morphology. These meshes are manufactured using a knitted textile structure, which is clearly observable in the SEM images shown in Figure 4. The knitting pattern contributes to their mechanical compliance and pore architecture, both of which play critical roles in tissue integration and inflammatory response.

Polytetrafluoroethylene (PTFE) is a fluoropolymer material characterized by its high electronegativity, low chemical reactivity, and pronounced hydrophobicity. These properties make it suitable for use in hernia repair meshes, particularly in minimizing tissue adhesion and chemical degradation within the body. However, its low porosity may limit tissue integration and cellular infiltration. Expanded PTFE (ePTFE) is a modified form of PTFE that retains the chemical inertness of the base polymer but incorporates a micro-porous structure. This expansion process enhances mechanical strength while allowing limited tissue ingrowth, which can improve implant stability [13,16]. ePTFE meshes are often used in applications requiring soft, conformable materials that still maintain adequate load-bearing capacity. Polyester (POL), on the other hand, is a hydrophilic material composed of multifilament carbon-based polymers. It is most commonly employed in open surgical hernia repair techniques. Polyester meshes offer notable advantages, such as strong integration with surrounding tissue due to their hydrophilic nature, which promotes cell adhesion and ingrowth. Additionally, they tend to result in minimal adhesion formation to surrounding organs, reducing the risk of postoperative complications [13,16].

Each material presents distinct mechanical and biological interactions with tissue, and the selection often depends on surgical technique, defect characteristics, and the desired balance between integration and complication risk.

Moreover, Surgipro™ presents the highest stiffness, potentially leading to complications as stress concentrations, while Neomesh SuperSoft^®^ is the most compliant, increasing the risk of failure because of mesh breakage [19]. Exploring modifications in hernia mesh designs, between ULTRAPRO™ Monocryl-Prolene Composite Mesh and Surgipro™, showed that modified mesh designs improve anchoring strength, reduce suture pull-through, and better distribute mechanical stress, reducing the risk of hernia recurrence [20]. Further study conducted on Vypro, Surgipro, Prolene, TiMesh, Optilene, Ultrapro, Dynamesh, and Proflex meshes suggests the use of lightweight, large-pore meshes for open inguinal hernia repair, as they offer better flexibility, reduced chronic pain, and improved tissue integration without increasing recurrence rates [21].

In addition to synthetic meshes, hybrid and biological meshes have been explored as alternatives in hernia repair. Hybrid meshes combine permanent synthetic materials with biologic components, offering structural support and enhanced biocompatibility [22], while biological meshes are derived from human or animal tissue, or synthesized biologic-based polymers, enabling native tissue regeneration [23]. However, while alternatives such as hybrid or coated meshes were included in FDA reports, according to the literature, there is no important difference in those meshes in comparison to other hernia meshes [6].

To overcome complications commonly associated with commercially available hernia repair meshes, such as inadequate mechanical compatibility and suboptimal load distribution, the Finite Element Method (FEM) emerges as a vital computational tool. FEM enables precise simulation of mesh-tissue interactions under physiological conditions, guiding the design of more effective implants. By optimizing mechanical performance and load transfer, FEM plays a crucial role in minimizing adverse outcomes and enhancing clinical success.

## 4. Mechanical Characterization and Finite Element Analysis

### 4.1. Mechanical Characterization of Soft Tissues in Hernia Repair

The abdominal wall is a dynamic and mechanically active structure, undergoing significant deformation due to breathing, movement, and intra-abdominal pressures. For the successful hernia mesh integration, the understanding and matching of the mechanical behavior of soft tissues and the mesh is pivotal.

In a study conducted by Hernández et al., the passive mechanical properties of the abdominal wall tissues were characterized through uniaxial tensile tests, with the investigation focused on the external oblique (EO), internal oblique (IO), transverse abdominis (TA), and rectus abdominis muscles of New Zealand White rabbits [24]. The samples were examined both as individual muscle layers and as composite tissues, in both the craneo–caudal and the perpendicular directions, revealing the anisotropic non-linear mechanical behavior of the abdominal tissues.

Additionally, the active mechanical behavior of abdominal wall muscles was explored under in vitro conditions [25]. The investigation was focused on the characterization of the force-generating capacity of muscle tissues when electrically stimulated. This way, it was aimed to understand the abdominal wall functionality in both physiological and pathological conditions for accurate modeling. The samples were harvested from adult male New Zealand White rabbits, including EO, IO, and TA muscles. The experiments were performed in physiological solution and involved applying cyclic length variations to the muscle strip. The tissue was activated during each cycle, and the resulting force was measured. The parameters obtained for each muscle were integrated into a finite strain formulation to simulate muscle activation, accounting for the tissue’s anisotropic properties, resulting in the model’s capability to predict the tissue’s anisotropic behavior and its impact on strain, stress, and the force generated during isometric contraction [25].

### 4.2. Hernia-Soft Tissue Simulation

Numerical models play a crucial role in understanding the biomechanics related to hernia repair by taking into account the variations in patient profiles, surgical techniques, and device characteristics. These methods have the potential to guide future device designs, enhance surgical procedure simulations, and support the personalization of surgeries, aiming to reduce recurrence rates [26]. In a study conducted by Fortuny et al., the internal oblique muscle was simulated, providing the most significant dynamic structure within the Hessert’s triangle [27]. This triangle includes both dynamically active and passive elements and is highly associated with the development of inguinal hernias, focusing on the transversalis fascia, a crucial structural element in the inguinal region, which examined under various conditions, demonstrates the impact of mechanical property variations on hernia formation. The data for the structure of the anatomical models was obtained from the Visible Human Project (VHP). Muscle contraction was modeled using the Hill-Maxwell model, combining one contractile and two elastic elements, with the contractile behavior being activated via a calcium-dependent function. A reference model of a healthy model with no pathology was established, with simulations repeated with parameters such as muscle and tendon Young’s modulus, tissue density, and Poisson’s ratio. Each variation’s effect on fascia strain was measured, and the results were visualized through strain maps, displacement diagrams, and parameter-strain graphs, allowing insight into hernia formation risks.

**Figure 5:**
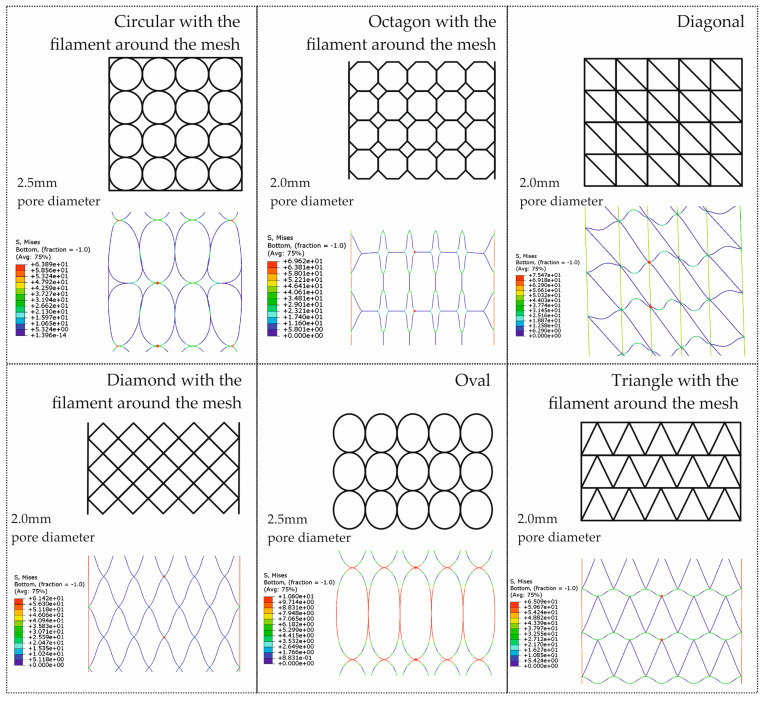
Finite Element Analysis (FEA) of six biodegradable mesh topologies with varying pore geometries under tensile loading. Simulations show differences in stress distribution and deformation behavior depending on pore shape. These models, originally developed for pelvic organ prolapse repair, are presented here to illustrate key mechanical insights that are also relevant for hernia mesh development. Adapted from [29,30].

Another model regarding the abdominal wall muscles, including the use of an electro-mechanical continuum approach for the modeling of muscle activation, revealed that fiber orientation and layered structure significantly affect contraction dynamics, with gradual fiber alignment observed during multi-layer contraction, and maximum stresses concentrated near fixed boundaries [25]. To assess the mechanical behavior of mesh-fascia fixation systems under dynamic loads, a combination of experimental testing and finite element modeling was developed, using porcine abdominal tissue, five mesh types, and five fixation methods, such as sutures, tacks, and glues, were subjected to impulse loads, while tensile tests provided material properties used to calibrate the FEM simulations. The strongest and most reliable fixation was exhibited from trans-abdominal sutures and ProTack connectors, while DynaMesh showed the highest resistance among the tested implants [28]. To explore the influence of pore geometry on mechanical performance under tension, finite element simulations were conducted on six mesh topologies with varying pore shapes, as shown in Figure 5. These results are adapted from our previously published studies [29,30], originally developed in the context of pelvic organ prolapse repair, and are presented here to illustrate general principles of pore deformation and stress distribution relevant to soft tissue mesh design.

Understanding these effects is essential not only for pelvic implants but also for the design and optimization of hernia meshes, where pore size and shape play a critical role in balancing mechanical strength, flexibility, and tissue integration.

A study testing surgical meshes using a finite element model combined with uniaxial tensile tests showed the Infinit^®^ demonstrating displacement and stress distributions closest to those of a healthy tissue [31]. The study investigated the mechanical behavior of three surgical meshes: Surgipro^®^, Optilene^®^, and Infinit^®^ through experimental testing and finite element (FE) simulations to evaluate their suitability for abdominal wall hernia repair. Specimens of each mesh type were prepared and soaked in Hanks solution for 24 h, and uniaxial tensile tests were performed in two orthogonal directions to assess anisotropy. To reproduce mechanical behavior, each mesh was assigned a hyperelastic constitutive model. More specifically, Surgipro^®^ was fitted with an isotropic Demiray SEF, while the anisotropic behavior of Optilene^®^ and Infinit^®^ was modeled using the Demiray-Holzapfel SEF. Following, FE simulations of the meshes were conducted, as well as an anatomically simplified FE model of a rabbit abdomen, including a hernia defect, where the surgical meshes were simulated [31].

To capture effectively the biomechanical impact of mesh repair, a 3D finite element model of the abdominal wall was developed with MRI data from a healthy subject. Following a hernia defect, as well as a surgical repair with intraperitoneal mesh placement, was simulated to compare healthy, herniated, and post-repair states under physiological intra-abdominal pressures, with the repaired abdomen exhibiting increased stiffness and slightly lower displacements than the healthy one [32].

A 3D multi-scale chemo-mechanical model of the abdominal wall was also introduced by Karami et al. to simulate muscle activation and intra-abdominal pressure (IAP), incorporating both active and passive muscle behavior [33]. Constitutive relations are derived from a strain energy function that accounts for chemical states, fiber anisotropy, and volumetric incompressibility, while muscle geometries were obtained from MRI data (BodyParts3D) and meshed in ABAQUS. Following, simulations of coughing in the supine position were performed, using calcium transients derived from EMG data for each muscle.

To enhance accuracy and real-time performance for soft tissue simulation, a hybrid modeling approach was developed, combining the finite element method with the particle-spring model (PSM) [34]. A triangular surface mesh was used to represent the soft tissue, with structural, bending, and shear springs incorporated to reflect mechanical behavior and preserve deformation continuity. Parameters for the particle–spring model were estimated using FEM-derived stress–strain relationships, considering non-linear, viscoelastic, and quasi-incompressible biomechanical properties of soft tissue, and validated against in vivo and in vitro data. The experimental data were obtained from previously published studies on porcine liver, and in simulation, the force-displacement responses were calculated based on user-applied input through a haptic device.

### 4.3. Synthetic Mesh

Prosthetic mesh failures in hernia repair are usually associated with complications, such as contraction, erosion, and organ perforation, which are influenced by mechanical interactions between the mesh, sutures, and surrounding tissue [35]. To influence those interactions, a biomechanical model was designed by Chanda et al., where vaginal tissue phantoms were fabricated using a custom elastomer mixture to match the mechanical behavior of human tissue. Samples of both the phantoms and Prolene^®^ meshes were prepared and tested under uniaxial tension at three different strain rates. A finite element (FE) model was created by reconstructing the prosthetic mesh structure from microscope images and modeling the sutures and tissue block in ANSYS APDL 17.1 FE software (Ansys Inc., Cannonsburg, PA, USA). Material behavior was captured using the Veronda–Westmann hyperelastic model for the tissue and mesh, while sutures were modeled as linear elastic materials.

For the simulation of the mechanical response of the commercial mesh available as DynaMesh^®^-IPOM under intra-abdominal pressure loading, two different constitutive modeling approaches were used [36]. The experimental characterization of the implant was made through uniaxial and biaxial tensile tests. Mechanical properties, such as stress–strain responses, were measured using a Zwick Roel machine combined with digital image correlation (DIC) techniques. A circular membrane geometry with a central hernia defect was modeled, and two Finite Element models were created; one with the dense material approach in MSC. Marc and the other with the GOH Hyperelastic anisotropic model in Abaqus. The simulations applied a pressure load that mimicked the intra-abdominal forces, and both models predicted the principal stress fields and displacements; and results were compared to experimental deflection data from a physical hernia model. Additionally, with material parameters from various biaxial tensile tests, both force- and displacement-controlled, seven model variants were created, with the equibiaxial force-controlled test being the most accurate constitutive model for simulating the prosthesis’ mechanical behavior under intra-abdominal pressure [37].

Moreover, a numerical model was developed by Wei He et al. to simulate the mechanical interactions between the abdominal wall and meshes post-repair, aimed to determine the most suitable mechanical properties of the meshes, focusing on elastic modulus and tensile strength [38]. The study modeled various hernia defects, including 20 mm and 40 mm defects located on the linea alba (LA20 and LA40) and the rectus abdominis (RA20 and RA40), where the findings indicated that the optimal mesh stiffness depends on the hernia’s location and defect size [38]. Another study suggests that synthetic, macroporous polypropylene meshes are the preferred choice for ventral hernia repair due to their lower complication rates, better tissue integration, and reduced recurrence compared to biologic meshes. The commercial synthetic meshes used in the study include medium-density, macroporous polypropylene mesh for open repairs and expanded polytetrafluoroethylene (Gore DualMesh Plus) for laparoscopic repairs. The biologic meshes examined include Strattice (porcine acellular dermal matrix) and Surgisis Gold (acellular porcine small intestine submucosa) [39].

The use of polypropylene hernia patches was explored by Liu et al., analyzing their biomechanical behavior through combined experimental testing and computational modeling [40]. The visco-hyperelastic constitutive model that was developed combined a quasi-linear viscoelastic (QLV) component and a hyperelastic Gasser–Ogden–Holzapfel (HGO) component. The finite element simulations were performed in the Abaqus 6.13–1 software to reproduce tensile and relaxation tests virtually. The model incorporated the measured fiber architecture, applied boundary conditions, and experimental loading paths, with the simulations showing great agreement with the experimental results.

Additionally, aiming to enhance tissue regeneration and reduce recurrence rates in hernia repair, the use of an electrically active hernia repair mesh was explored by Mosier et al., incorporating piezoelectric materials, such as polyvinylidene fluoride (PVDF) [41]. The model was based on typical human abdominal tissue layers, and the mesh was modeled as a cylindrical network mimicking polypropylene surgical meshes. Electric field intensities were calculated across different tissue layers. Vertical and cross-sectional slices were analyzed to determine field magnitudes at critical locations (see Table 2) [17]. It was shown that the retromuscular placement produced the highest fields within the rectus abdominis muscle and connective tissue layers, which are essential for hernia healing, while minimizing electric field exposure to the small intestine.

### 4.4. Constitutive Laws

Constitutive relations are the equations that describe a material and its response to loading [42]. Three general principles determine the constitutive laws of materials: determinism, local action, and objectivity. For biological tissues, the stress at a given material point depends on the deformation of the point at the actual time, as well as at all previous times [43]. The general constitutive law for such tissue is (Truesdell and Noll) Equation (1):

S(t) = Se(C(t)) + I∞ s = 0 G(t − s); C(t)(1)

where S is the second Piola–Kirchhoff stress tensor, C is the right Cauchy–Green strain tensor, I is a functional representing the history of G(t − s) = C(t − s) − C(t) and Se(C(t)) is an equilibrium term. The stress S and strain C are two symmetric second-order tensors. The notation in (1) means that G(t − s) is a variable and C(t) is a parameter. A rearrangement of the constitutive law is then made according to the time scale, proposing the following general viscoelastic description for soft biological tissues Equation (2):

S = SeC(t) + Sv Ċ(t); C(t) + ∞ δ G(t − s), s; C(t) ds(2)

with Sv the second Piola–Kirchhoff viscous stress tensor and Ċ the strain rate tensor [43].

Assuming that abdominal wall tissues behave as transversely isotropic hyperelastic materials, the strain energy function was split into volumetric and isochoric components. The isotropic part was decomposed into isotropic and fiber-related contributions. The model was calibrated using experimental tensile test data and successfully reproduced the mechanical responses of different abdominal tissues under tension, offering a reliable and computationally efficient basis for simulating abdominal wall mechanics [44]. In the formulation, both contributions to the strain energy were expressed using convex polynomial functions of the relevant strain invariants. The isotropic component was modeled as a function of the first invariant I_1_, while the fiber contribution was based on the pseudo-invariant I_4_, representing the squared stretch in the fiber direction. The resulting strain energy density functions are given by Equation (3):

Ψ_iso_ = C_1_(Ī_1_ − 3) + C_2_(Ī_1_ − 3)^2^ and Ψ_fib_ = C_3_(Ī_4_ − 1)^2^ + C_4_(Ī_4_ − 1)^4^ (3)

where C_1_, C_2_, C_3_ and C_4_ are positive constants. This structure ensures smooth mechanical response and numerical stability, making the model suitable for finite element simulations involving both passive and active abdominal wall tissues under moderate deformation levels.

To optimize the reproduction of the mechanical response of hernia meshes fixed in the abdominal wall, three hyperelastic models are evaluated: Gasser-Ogden-Holzapfel, Demiray-Holzapfel, and Neo-Hookean-Humphrey-Yin. Each model includes strain energy components based on invariants of the right Cauchy-Green tensor and is calibrated using different bi-axial test modalities. The objective function differs depending on the anisotropy of the mesh implant. In anisotropic mesh implants, it minimizes the angular error between experimental and simulated positions of peak reaction forces. In isotropic, it minimizes the variance of reaction forces across fixation points [45].

Among the models evaluated, the Gasser–Ogden–Holzapfel (GOH) law proved to be the most adaptable across both anisotropic and isotropic cases, offering uniform stress distributions and minimal deviations from experimental data. The strain energy function for this model, incorporating both isotropic and anisotropic effects, is defined as Equation (4):

(4) $$\Psi \;=\;C_{10}(I_{1}\;-\;3)\;+\;\frac{k_{1}}{{2k}_{2}}\sum_{i=4,6}\{\exp\;[{k_{2}(\kappa I_{1}\;+\;1\;-\;3\kappa )}^{2}]\;-\;1\}$$

Here, I_1_ is the first invariant of the right Cauchy–Green deformation tensor, while I_4_ and I_6_ are pseudo-invariants describing stretches along preferred directions of anisotropy. The constants C_10_, k_1_, and k_2_ are material parameters, and κ∈[0, 1/3] is the fiber dispersion factor, with κ = 1/3 representing isotropy. This model’s ability to reflect both uniform and directionally dependent behaviors under simulated intra-abdominal pressure makes it highly suitable for numerical modeling of implanted surgical meshes.

## 5. New Approaches for Hernia Mesh Implants

### 5.1. Methods

#### 5.1.1. Three-Dimensional Printing

With the development of 3D printing technology, new possibilities in healthcare have opened, allowing for customized and precise solutions to various medical challenges. Three-Dimensional printing was originally developed for industrial manufacturing but is now widely used in hospitals to create medical devices, prosthetics, and surgical instruments, including those used in hernia repair. This technology enables rapid prototyping, allowing healthcare professionals to develop and test new designs faster than traditional manufacturing methods, allowing the production of patient-specific models and implants [46,47].

Additionally, with the help of medical imaging techniques, such as MRI and CT scans, customized anatomical models can be created, improving surgical planning and patient outcomes [46,47]. In a feasibility study conducted by George et al., the design and fabrication process of 3D-printed surgical instruments were explored, emphasizing their use in surgical procedures, such as inguinal hernia repair [48]. The researchers designed and printed a surgical instrument set, including needle drivers, hemostats, forceps, scalpel handles, and retractors, with the final version being capable of occluding the hernia sac and effectively manipulating tissue planes. Additionally, 3D printing combined with electrospinning is presented to create biological composite scaffolds from polymer fibers and collagen, which can support hernia repair and abdominal wall reconstruction, providing mechanical stability and promoting cell attachment, as well as tissue regeneration [49,50]. In vivo tests showed that using 3D printing, there was minimal inflammatory response with successful defect closure, confirmed through X-ray and ultrasound imaging, which indicated good integration without severe exudation [51]. More specifically, as reported by Erwin et al., the 3D printing process enabled the creation of a hernia mesh that allowed tissue to anastomose into the mesh’s pores. This structural feature facilitated connective tissue proliferation and neovascularization, which helped close the hernia defect. The integration of tissue into the mesh implant, without pathological changes in the surrounding muscles, indicates that the 3D-printed porous architecture contributed to a minimal inflammatory response.

An alternative to traditional PP meshes for hernia repair is 3D-printed medial-grade polycaprolactone (mPCL) scaffolds, fabricated using melt electrowriting (MEW) technology, and designed to mimic the biomechanical behavior of native soft tissues, improving flexibility and strength, suggesting MEW-based biodegradable meshes’ promising used in contrast to conventional synthetic implants due to their biocompatibility and adaptability to dynamic soft tissues [46,52,53]. In addition to the scaffolds, the use of a bi-layered composite patch developed using 3D printing and electrospinning was also explored, providing antibacterial and antiadhesive properties. The composite patch consisted of an antibacterial layer, which prevents infections and promotes tissue regeneration, and an antiadhesive one, which reduces postoperative adhesions [54].

#### 5.1.2. Electrospinning

Electrospinning is a versatile technique that produces nanofibrous materials with high surface area, porosity, and tunable mechanical properties, making it especially suitable for biomedical use. Its ability to mimic the structure of the extracellular matrix supports applications in tissue engineering, wound dressings, and controlled drug release [55,56].

In a study conducted by Kaya et al., a dual-component mesh was developed by electrospinning an absorbable PGS/PCL nanofibrous layer onto a nonabsorbable polycarbonateurethane (PU) mesh, creating a composite structure suitable for hernia repair [57]. The electrospun layer effectively acted as a barrier to prevent visceral adhesion, while maintaining biocompatibility and supporting cell proliferation. The electrospinning technique resulted in a final product with uniformly integrated layers, as revealed by scanning electron microscopy [57].

Additionally, electrospun fibrous membranes were explored and shown to be effective in reducing tissue adhesion in inguinal hernia repair by forming a physical barrier between the mesh and surrounding organs [58]. The membranes showed enhanced biocompatibility, supporting fibroblast growth while minimizing inflammatory response. These findings suggest that electrospinning offers a protective strategy to improve the safety and functionality of hernia mesh implants.

#### 5.1.3. Melt Electrowriting (MEW)

MEW is an advanced additive manufacturing technique with the ability to produce highly precise micro- and nanofiber scaffolds, widely used in the biomedical field. The technology is based on the electrohydrodynamic process of polymer melts, with the use of a high-voltage electric field to create fibers with diameters as small as sub-microns [59].

MEW provides exceptional control over fiber deposition, allowing the creation of complex, patient-specific scaffolds that mimic the structural and mechanical properties of native tissues [60]. The flexibility of MEW lies in its ability to utilize biodegradable polymers such as polycaprolactone (PCL). PCL is an FDA-approved material known for its excellent biocompatibility and long degradation periods. Its properties make it ideal for fabricating scaffolds designed for tissue engineering and medical implants [61]. Key parameters in MEW that influence fiber diameter and scaffold morphology are the melt temperature, collector speed, tip-to-collector distance, as well as flow rate. At the same time, studies indicate that optimizing these parameters ensures structural integrity and application-specific functionality of MEW-fabricated scaffolds [61].

MEW has emerged as a promising technology for developing biodegradable and biocompatible meshes ideal for POP and hernia repair. Unlike traditional polypropylene meshes, MEW allows the production of structures with precise geometries, such as sinusoidal and auxetic designs that mimic the mechanical properties of tissues. These meshes demonstrate improved porosity and mechanical stability under tensile loads, addressing limitations related to conventional surgical implants [62]. Studies have shown that MEW-produced PCL mesh implants achieve adjustable mechanical properties by varying parameters like fiber amplitude and wavenumber. This allows the development of patient-specific implants, reducing the risk of complications and improving post-operative outcomes. Additionally, the direct-writing capability of MEW enables the precise stacking of fibers, resulting in scaffolds that support cell attachment and proliferation, which is crucial for effective tissue regeneration [60,62].

Ren et al. studied the development of biodegradable composite meshes using MEW to address the limitations of traditional polypropylene meshes for POP repair, focused on PCL and polyethylene glycol (PEG) composites, fabricated in 90:10 and 75:25 ratios, which were evaluated for mechanical properties, degradation rates, and biocompatibility [63]. The results showed that increasing PEG content allowed for controlled degradation, with the 75:25 composite degrading more quickly and forming hollow structures under physiological conditions. Additionally, the mechanical strength and stiffness of the PCL/PEG meshes were significantly improved, with the 75:25 composition achieving a 127% increase in tensile strength compared to PCL alone [63]. For further improvement in functionality, the meshes were coated with the antibiotic azithromycin, which effectively restrained bacterial growth for up to 14 days. However, initial cytotoxicity was observed in drug-loaded meshes, though pre-release in phosphate-buffered saline mitigated this effect, enabling cell attachment and proliferation over time. This study highlights the MEW-fabricated PCL/PEG meshes as a promising alternative for safer and more effective treatments with mesh implants [63].

### 5.2. Types of Mesh Implants

#### 5.2.1. Biologically Based Materials

Surgical meshes have significantly reduced recurrence rates compared to sutures alone, with nonabsorbable synthetic meshes, such as polypropylene, remaining the clinical standard for hernia repair due to their strong mechanical properties. However, they are prone to various complications, such as complications like chronic inflammation, pain, infection, and adhesions. In contrast, biologically based materials present advantages in biocompatibility and bioresorbability, making them a promising alternative to synthetic meshes [15]. Recent innovations include coatings with chitosan, polydopamine, and zwitterionic polymers, nanofiber technologies, and fully absorbable polymer meshes, such as polyglycolic acid (PGA) and poly-4-hydroxybutyrate (P4HB), which degrade over time to promote tissue regeneration and enhance antimicrobial properties [1,64].

A bioresorbable silk fibroin (SF) mesh is shown to offer superior mechanical properties, such as optimized tensile strength, flexibility, and lower stiffness, reducing post-surgical complications [65]. The SF layer can be created using electrospinning and then adhered to the polypropylene (PP) mesh using fibrin hydrogel, forming a hybrid biological-synthetic mesh [66]. In a study conducted by Bokhari et al., hand-knitted silk meshes were developed, coated with biodegradable polymers and natural extracts, offering mechanical strength and enhanced biocompatibility, as well as antibacterial properties. The polymer coatings significantly increased the tensile modulus, tensile strength, and reduced strain percentage, suggesting improved durability for surgical applications [67]. An example of a bio-inspired polydopamine (PDA) coating was developed by Sanbhal et al., improving surface wettability, as well as enhancing the ability of the mesh to absorb and retain antimicrobial agents, reducing infection risks post-implantation [68].

Additionally, the combination of polypropylene meshes with hydrogels, such as poly(N-isopropylacrylamide) (PNIPAAm) hydrogel and dopamine-functionalized polysaccharide hydrogel (OCMC-DA/CMCS), reduced cell adhesion, collagen deposition, as well as complications such as chronic pain [69,70]. Another alternative is nanofiber-containing polypropylene-based composite meshes, more specifically electrospun nanofibrous membranes (NFM) made from poly(lactic-co-glycolic acid) (PLGA) and polycaprolactone (PCL) integrated into PP meshes to reduce adhesion, due to their biocompatibility, while maintaining great mechanical support [71], or poly(glycolide-ε-caprolactone) (PGCL) integrated into polypropylene (PP) using wrap-knitting technology, which is shown to demonstrate superior tensile strength and bursting resistance for hernia repair [72]. A multifunctional hernia repair biopatch was also developed by combining 3D printing and coaxial electrospinning exhibited superior mechanical strength and antibacterial effects, because of its ciprofloxacin (CIP)-loaded polycaprolactone (PCL)/gelatin (Ge) scaffold, and improved tissue regeneration and wound healing, due to its electrospun nanofiber layer made of PCL/Ge/κ-carrageenan (κ-C) [73].

#### 5.2.2. Smart Materials

Smart materials are increasingly shaping innovation in healthcare, offering functionalities that range from responsive materials to advanced medical devices. According to Ghosh (2024), smart materials have a unique ability to alter their properties, such as Young’s modulus, in response to external stimuli like temperature, pressure, PH, and magnetic fields. Because of their adaptability, they are suitable for a variety of biomedical applications, especially in fields like orthopedics or cardiovascular treatments [74,75]. Smart implants are used to replace damaged tissues or organs, monitor patient status, and actively participate in treatment, with biodegradable implants eliminating the need for surgical removal [76].

Smart biopolymers have also been applied in the field of tissue engineering, and more specifically, thermo-responsive polymers are widely used as substrates to support cell growth and proliferation, by controlling the adhesion and release of cells on their surface [77]. An example of a thermo-sensitive polymer is PNIPAAm, which is used in drug delivery systems. Forming injectable hydrogels that gel at body temperature allows site-specific delivery of drugs or cells.

Additionally, the polyethylene glycol (PEG) and poly(methacrylic acid) (PMAA) graft copolymer (PMAA-g-PEG) is a pH-responsive material that swells in response to increasing pH due to the ionization of its carboxylic groups. This behavior makes it suitable for targeted drug delivery, such as the intestinal release of calcitonin, where the polymer remains stable in the acidic stomach and releases the drug in the more basic environment of the small intestine [77]. Another application of pH-responsive smart materials for targeted drug delivery includes poly(acrylic acid) (PAA)-based nanoparticles, particularly in cancer therapy, where the acidic tumor environment triggers the release of therapeutic agents [78]. In drug delivery, photo-responsive hydrogels are also of great interest. Hydrogels composed of cyclodextrin or azobenzene modified dextran are being explored, since they present advantages, because the light stimulus can be applied and removed very quickly, allowing rapid control over the hydrogel’s behavior, and with great accuracy [79].

Materials for hernia repair have also evolved from prosthetic meshes to smart biomaterials with adaptive properties, enhancing tissue integration. The advancements are focused on hydrogels, cryogels for better cell adhesion, inflammation control, and biodegradability, as well as electrospun fibrous membranes, and 3D-printed scaffolds, for improved scaffold functionality [80,81]. Typically, in the case of hernia, the goal is to promote integration with host tissue on the parietal side (e.g., muscle or fascia) while minimizing adhesions to visceral organs to prevent complications such as bowel obstruction or chronic pain. Therefore, controlled and site-specific cell adhesion is key—enhancing cell adhesion on the side facing the abdominal wall can improve mesh incorporation, while the opposite side is often designed to resist cellular adhesion to prevent visceral adhesions.

Moreover, their ability to maintain moisture balance and reduce inflammation improves healing, and techniques such as self-assembly, molecular imprinting, and electrospinning allow for the development of smart hydrogels with self-healing properties, and enhanced tissue interactions [82,83]. Additionally, piezoelectric and electroactive materials exhibit enhanced cell growth and tissue remodeling, contributing to more effective and adaptive hernia repair strategies [84]. These materials generate electrical activity in response to mechanical stress, mimicking the bioelectric signals that influence proliferation, differentiation, and migration, participating actively in the healing process by providing localized electrical stimulation [85].

An approach in hernia repair includes modifying PP surfaces with silanization and the attachment of the HBII-RGD molecule, which is a fibronectin-derived peptide designed to promote cell adhesion. With this biofunctionalization strategy, the cellular responses were exhibited to be enhanced for primary abdominal fibroblasts, as experimental studies demonstrated that functionalized PP surfaces significantly improved fibroblast adhesion, spreading, and cytoskeletal organization [86].

#### 5.2.3. Drug-Eluting Meshes

Various studies have shown that the presence of a foreign material in the human body, such as a surgical mesh or other implantable device, significantly increases the possibility of infection [87]. Synthetic meshes provide strong mechanical support but are prone to inflammation, biological meshes may lack durability, and composite meshes aim to balance adhesion prevention with tissue integration [88]. A promising advancement to avoid bacterial contamination is the integration of drug-eluting meshes, which deliver antibiotics, anti-inflammatory agents, or growth factors to improve healing and reduce complications around the surgical site [87,88]. Various alternatives have been developed to reduce bacterial colonization and promote tissue regeneration, such as hydrogel coatings, nanofiber modifications, and antibiotic-infused polymer layers [88].

Hydrogels provide an effective physical barrier to adhesion formation and serve as a sustained-release drug delivery system, and combined with sirolimus, which has anti-inflammatory and antiproliferative properties, results in a mesh with enhanced adhesion prevention while maintaining local drug release without systemic absorption [89]. The hydrogel-impregnated polypropylene mesh exhibited significantly reduced intra-abdominal adhesion formation, with the adhesion surface area being reduced from 100.0 ± 0% in the plain mesh control to 18 ± 8% in the sirolimus/hydrogel-impregnated mesh (*p* < 0.001) [89]. A polypropylene mesh was also combined with an anti-adhesion layer of poly(vinyl alcohol) (PVA) hydrogel and a liposome-based drug delivery system for controlled rapamycin release, formed using freezing-thawing processing cycles (FTP), creating a composite hernia mesh. The composite mesh demonstrated a significant reduction in postoperative adhesion in a rat model compared to the unmodified PP mesh, with adhesion scores of 1.37 ± 0.75 and 3 ± 0.71, respectively, while histological analysis showed decreased inflammation and a looser fibrous tissue structure, indicating improved biocompatibility and reduced adhesion formation [90]. Another hydrogel used as a coating for hernia meshes is a thermo-responsive hyaluronic acid-poly(N-isopropylacrylamide) (HApN) hydrogel, forming a stable gel upon reaching body temperature, allowing this way the controlled delivery of antimicrobial agents without significantly altering the mechanical properties of the mesh [91]. The drug loading could include either antibiotics, such as gentamicin and rifampicin, or antiseptics, such as chlorhexidine, which were shown to effectively inhibit the growth of biofilm-forming bacteria, including Staphylococcus aureus and Escherichia coli [91,92].

Additionally, poly(ε-caprolactone) (PCL) was explored as a coating or part of a coating of polypropylene meshes, providing a smooth, homogeneous shell that allows drug release without significantly altering the biomechanical properties of the mesh [87]. Combined with ofloxacin, it exhibited strong antibacterial activity, reduced cell adhesion, biofilm formation, and maintained proper cell viability [87,93]. To reduce bacterial colonization and tissue adhesion, apart from PCL, the mesh was coated with nanofibrous mats incorporating carboxyethyl chitosan (CECS) and polyvinyl alcohol (PVA) containing ofloxacin for antibacterial properties [93].

### 5.3. Hernia Regeneration Attempts

Traditional synthetic meshes, while providing strong mechanical support, are often associated with complications such as chronic inflammation and limited tissue integration. Regenerative approaches aim to restore functional tissue by creating a biological environment that promotes healing rather than scar formation.

Fully absorbable mesh implants made from poly-4-hydroxybutyrate (P4HB) have been explored in a rat model of abdominal wall defects, where they supported extensive tissue remodeling. Histological analyses revealed significant fibroblast infiltration, neovascularization, and organized collagen deposition over time, suggesting that degradable meshes can serve not only as temporary scaffolds but also as active participants in the regenerative healing process [94].

Another approach involves a dynamic 3D scaffold (ProFlor), designed to actively stimulate tissue regeneration in inguinal hernia repair. MRI and histological analyses in a porcine model showed the formation of vascularized connective tissue and muscle-like structures within the scaffold. These findings suggest that the scaffold’s mechanical behavior contributes to a regenerative microenvironment that helps prevent tissue degeneration and supports functional restoration [95].

Additionally, biological meshes derived from decellularized porcine small intestinal submucosa (SIS) have shown promise in promoting regeneration. In a porcine ventral hernia model, SIS meshes led to significantly increased neovascularization and cellular infiltration, as assessed by a semi-quantitative histological scoring system. The preserved extracellular matrix (ECM) and residual bioactive components appear to play a key role in modulating host cell behavior and supporting tissue ingrowth and remodeling [96].

Collectively, these regenerative strategies reflect a shift from passive structural support toward biologically active solutions that engage host tissues in the healing process. Although further clinical validation is needed, the integration of degradable materials, bioactive scaffolds, and tissue-responsive designs represents a promising direction for improving long-term outcomes in hernia repair.

## 6. Future Perspectives and Market Analysis

### 6.1. Future Perspectives

Mechanical compatibility between the implanted mesh and the abdominal wall is a fundamental aspect of achieving long-term success in hernia treatment. However, traditional mesh implants, especially synthetic ones, often fail to meet these requirements because of several mechanical limitations [97].

One of the key mechanical issues is the high stiffness and inflexibility of the mesh implants, which restrict the natural motion of the abdominal wall, leading to excessive scarring and patient discomfort. Another common problem is the mismatch between the implant’s and the native tissue’s elasticity. The abdominal wall is elastic and highly dynamic, and many mesh materials fail to replicate this behavior. According to Ibrahim et al., an optimal mesh should exhibit higher biomechanical strength and greater elasticity than the abdominal wall, resulting in hernia recurrence due to the higher elasticity of the mesh implants. Additionally, high initial tensile strength does not guarantee long-term durability, as it was found that some materials lose mechanical integrity under repeated physiological loading, leading to unpredictable biomechanical behavior of the meshes after implantation [97,98].

The successful integration of auxetic properties and negative thermal expansion behavior in composite meshes presents a promising approach for designing advanced materials with applications in both structural and biomedical fields, offering significant advantages in managing thermal stress and enhancing the performance of devices such as hernia meshes and stents [99].

Auxetic materials are characterized by their negative Poisson’s ratio (NPR) and their property of becoming thicker when stretched and thinner when compressed, offering advantages in biomedical applications, impact-resistance materials, and engineered structures [100]. An application of auxetic materials in biomedical engineering is in tissue scaffolding, with Warner et al. (2024) demonstrating 3D-printed auxetic scaffolds for muscle and tendon regeneration, fabricated via Dynamic Optical Projection Stereolithography (DOPsL). These scaffolds allow for precise control over mechanical properties, providing enhanced tissue integration [101]. Auxetic pore geometries also offer significant advantages in surgical meshes, as they expand laterally under tension, maintaining pore openness and mechanical stability under physiological loads, and preventing pore collapse [102].

**Figure 6:**
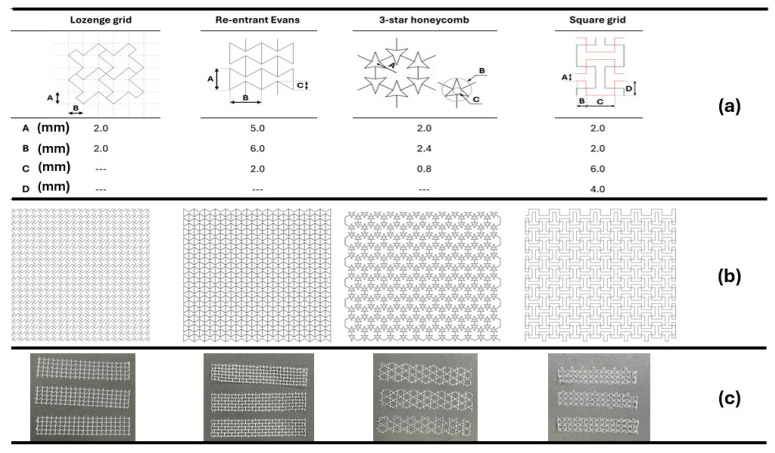
Auxetic mesh designs for biomedical applications. (**a**) Unit cell dimensions of four distinct auxetic geometries (**b**) Corresponding geometric patterns illustrating the pore architecture of each design. (**c**) Photographs of fabricated auxetic mesh samples used in uniaxial tensile testing. Adapted from [103].

To illustrate the design potential of auxetic geometries in mesh implants, Figure 6 presents four auxetic mesh topologies—lozenge grid, re-entrant Evans, 3-star honeycomb, and square grid—showing (a) unit cell dimensions, (b) geometric patterns, and (c) fabricated samples through MEW [103]. These structures demonstrate how pore geometry can be engineered to achieve tailored mechanical behavior and improved compatibility with dynamic tissues.

Additionally, because of the auxetic properties of several biological tissues, such as skin, arteries, and the annulus fibrosus of intervertebral disks, their recreation within scaffolds influenced stem cell fate, promoting differentiation into neural and vascular lineages while increasing osteoblast and chondrocyte proliferation under dynamic loading conditions. Therefore, auxetic structures were included in biomedical implants, such as artificial intervertebral disks and hip implants [104]. In a study conducted by Kolken et al., the fatigue performance of additively manufactured (AM) auxetic structures made from commercially pure titanium (CP-Ti) was explored, showing that auxetic meta-biomaterials outperform many conventional biomaterials in cyclic loading due to their high energy dissipation and superior mechanical stability. Their ability to restore bone-implant contact along a hip stem’s lateral side is shown to help reduce implant failure risks by promoting bone compression and remodeling, improving implant longevity [105].

The current AM methods for manufacturing auxetic structures in biomaterials include metallic and polymeric ones. For metallic auxetic structures, with the most reported being Ti64 (Ti-6Al-4V), and then stainless steel and CoCrMo, the most widely used methods are laser power bed fusion (LPBF) and electron beam melting (EBM) [106]. Polymeric auxetic structures include PLA being the most reported, polyethylene glycol diacrylate (PEGDA), thermoplastic polyurethane (TPU), acrylonitrile butadiene styrene (ABS), and PCL, and two widely adopted methods include fused deposition modeling (FDM) and stereolithography (SLA) [106,107].

Looking ahead, the clinical translation of innovative mesh designs—particularly those incorporating auxetic geometries and biofunctional materials—must be critically evaluated not only in terms of mechanical innovation but also in light of patient safety, regulatory standards, and long-term clinical performance. Future research should focus on the comparative effectiveness of new mesh strategies in reducing complications such as chronic pain, infection, and poor integration. Innovation in this field must be driven by robust preclinical and clinical validation, aligning with current surgical guidelines and prioritizing features such as enhanced biocompatibility, reduced inflammatory response, tissue-specific mechanical adaptability, and ultimately, improved patient-reported outcomes. Only through this integrated approach can the next generation of hernia meshes meet both engineering excellence and clinical relevance.

### 6.2. Market Analysis

The global market for hernia mesh implants has expanded significantly over the past decade, driven by the increasing incidence of hernias, greater adoption of minimally invasive surgical techniques, and the continuous development of advanced biomaterials. Despite this growth, cost-effectiveness remains a major limiting factor, particularly in the choice of mesh type and fixation method [108].

Notably, biologic meshes—although associated with reduced infection rates and improved tissue integration—are substantially more expensive than synthetic alternatives. This price difference limits their widespread use in routine procedures, where synthetic meshes remain the economically preferred option [108].

In terms of market size, the global hernia repair market was valued at approximately USD 3.8 billion in 2022 and reached USD 4.1 billion in 2024. It is projected to grow at a compound annual growth rate (CAGR) of 4.2% from 2024 to 2029, reaching an estimated USD 5.1 billion by the end of that period. Key drivers of this growth include an increasing number of hernia repair procedures, broader use of mesh in surgeries, innovations in product design, a growing aging population, expanded reimbursement coverage, and the proven effectiveness of mesh-based repair techniques [109].

These trends underscore the urgent need for next-generation mesh technologies that combine enhanced biocompatibility, mechanical performance, and infection control with affordability and scalability. Innovations such as biodegradable polymers, drug-eluting systems, and computationally optimized designs have the potential to reduce long-term complications and overall healthcare costs, making them particularly attractive for broader adoption in both public and private healthcare systems [109].

### 6.3. Patent Analysis

Recent patents in hernia mesh development reflect a growing trend toward improved biocompatibility, patient comfort, and ease of surgical application.

One innovation involves the design of a calendered, thin-profile surgical mesh implant made from poly-4-hydroxybutyrate (P4HB) or its copolymers. This design maintains high burst strength while offering a smoother surface and reduced bulk, making it suitable for both hernia and pelvic organ prolapse repair (US10874498B2) [110].

Another patent explores the use of 3D printing to fabricate customizable hernia patches with tunable porosity, elasticity, and mechanical strength, aiming to reduce the foreign body sensation and streamline the manufacturing process (CN107756781B) [111].

A third example introduces a single-sheet electrospun nanofibrous mesh with a highly porous 3D architecture. Designed to degrade after tissue integration and scar formation, this approach seeks to minimize long-term discomfort while supporting effective healing (US20230149147A1) [112].

Lastly, advances in bioadhesive technologies offer alternative fixation strategies. One patent proposes a catechol-based hot-melt adhesive combining caffeic acid derivatives with synthetic thermoplastics. This heat-activated adhesive allows the mesh to bond to tissue without mechanical anchors, potentially reducing surgical trauma (WO2021059272A1) [113].

Together, these patents indicate a clear shift toward customized, biointegrative, and minimally invasive mesh solutions, underscoring the industry’s commitment to improving clinical outcomes through material science, fabrication innovation, and functional design.

## 7. Discussion

The development of hernia mesh implants has undergone considerable transformation in recent decades, evolving from simple synthetic scaffolds to complex, multifunctional systems designed to promote tissue regeneration while minimizing postoperative complications. Traditional materials, such as polypropylene (PP), remain widely used due to their mechanical robustness, but their association with complications—including foreign body response, infection, and chronic pain—has motivated the search for alternative solutions [6]. Biological meshes, although offering improved biocompatibility, may lack sufficient mechanical strength and present issues related to high cost and variability [15,108].

To address these limitations, recent advances in biodegradable and bioactive materials, such as polycaprolactone (PCL), poly(glycolic acid) (PGA), and hybrid systems, have shown potential to improve healing and integration [8]. These materials aim to strike a balance between structural support and controlled degradation, allowing host tissue to regenerate gradually as the implant resorbs. The choice of material must be aligned with the specific clinical context—whether for temporary reinforcement or permanent structural support.

Smart materials, capable of responding to external stimuli such as temperature, pH, or pressure, are a promising new frontier [75]. While most examples remain at the proof-of-concept stage, their integration into hernia repair systems may allow for on-demand drug delivery, dynamic stiffness modulation, or antibacterial response, potentially reducing complications like infection or fibrosis [76,77,79]. However, for clinical adoption, these materials must meet strict biocompatibility and long-term safety standards, and their functional performance must be validated in preclinical and clinical settings.

Furthermore, the introduction of auxetic geometries offers a mechanical innovation that complements material advances. Auxetic meshes, with a negative Poisson’s ratio, expand laterally when stretched, helping to maintain pore openness and improve conformability to tissue dynamics [100]. These properties can reduce the risk of pore collapse and improve load distribution, potentially minimizing pain and tissue damage [101,102]. While their use in hernia repair is still largely theoretical, findings from related fields such as orthopedics and cardiovascular implants suggest strong potential [105]. Additive manufacturing techniques like FDM, SLA, and LPBF make such designs increasingly feasible for biomedical applications [106,107].

Alongside materials and geometry, computational modeling—particularly FEM—has emerged as a powerful tool to simulate the behavior of meshes under physiological loads [38,40]. FEM allows the prediction of stress concentrations, deformation patterns, and implant-tissue interactions, guiding the optimization of both material selection and geometry. Although underutilized clinically, FEM holds promise for personalized mesh design based on patient-specific anatomy derived from medical imaging [46,47]. Coupled with experimental validation and inverse modeling, FEM could pave the way for predictive tools in pre-surgical planning.

Despite these technological advances, the translation into clinical practice remains a major challenge. Very few of the innovations discussed—smart meshes, auxetics, biodegradable scaffolds—have been fully evaluated in terms of long-term safety, cost-effectiveness, and real-world outcomes. Future research must prioritize not only mechanical and biological performance but also comparative effectiveness, considering endpoints such as infection rate, recurrence, chronic pain, and patient satisfaction.

Moreover, innovation should be aligned with clinical guidelines and surgical workflows, integrating feedback from clinicians early in the design process. Ultimately, mesh design should aim for enhanced biocompatibility, reduced foreign body response, improved tissue regeneration, minimized adhesion formation, and adaptability to individual patient needs, balancing innovation with practical feasibility.

## 8. Conclusions

This review examined the evolving landscape of hernia mesh design, encompassing advances in materials science, mechanical modeling, manufacturing techniques, and biologically inspired strategies. A broad spectrum of approaches is currently under investigation—from synthetic and biologically derived meshes to smart, stimuli-responsive systems, drug-eluting materials, auxetic geometries, and computationally optimized structures. While each of these domains offers distinct advantages, they must not be considered in isolation. The clinical utility of a mesh arises not from a single feature, but from the integration of multiple properties—mechanical strength, biocompatibility, degradation behavior, and adaptability to anatomical and functional requirements.

The guiding principle that emerges from this analysis is the shift from static, one-size-fits-all devices to dynamic, patient-tailored solutions. These new-generation implants aim to interact intelligently with the host environment—supporting tissue regeneration, resisting infection, and minimizing chronic inflammatory responses. This transformation is not merely technological; it reflects a broader, interdisciplinary transition toward evidence-based, personalized surgical care.

The integration of computational modeling, particularly Finite Element Modeling (FEM), offers a significant step toward the rational design of meshes adapted to individual biomechanics. FEM allows for simulation of load distribution, tissue deformation, and implant–tissue interaction under realistic physiological conditions. When combined with imaging data and inverse modeling, it opens the path to personalized, pre-surgical planning—a major evolution in the field.

Smart materials and auxetic designs provide further avenues for innovation. Thermo- and pH-responsive hydrogels, drug-loaded polymers, and electroactive scaffolds enable meshes to respond to local environmental stimuli, potentially reducing infection and improving healing. Auxetic geometries, with their negative Poisson’s ratio, improve conformity to dynamic tissues and may help reduce complications such as pore collapse and mechanical mismatch. However, these technologies remain in the experimental phase, and systematic clinical validation is urgently required to assess long-term performance and safety.

Importantly, innovation must not outpace clinical evidence. The most promising hernia meshes of the future will be those that align engineering performance with clinical priorities—reducing recurrence, minimizing pain, lowering infection rates, and improving quality of life. To realize this vision, several steps are essential:

- Rigorous in vivo and long-term clinical studies evaluating safety and efficacy;
- Greater integration of FEM and 3D design tools into early-stage development and surgical planning;
- Comparative effectiveness studies that benchmark new solutions against current standards of care;
- Development of regulatory frameworks and surgical guidelines that support innovation while ensuring patient safety;
- Continuous interdisciplinary collaboration between engineers, materials scientists, clinicians, and regulatory bodies.

In conclusion, the future of hernia mesh technology lies not in isolated innovation, but in strategic, systems-level integration. By aligning advanced materials, smart design, and clinical translation within a common framework, the next generation of hernia implants can move from concept to clinic—offering personalized, high-performance, and safer solutions for one of the most common surgical procedures worldwide.

## Figures and Tables

**Table 2:** Mechanical properties of commercially available meshes [17].

Mesh Name	Type	Direction	T_0_ (MPa)	T_eq_ (MPa)	ΔΤ (%)	E_0_ (MPa)	E_eq_ (MPa)
MicroVal 2D Mesh	PP	L	0.238 ± 0.042	0 (t = 1400 s)	100.00	4.76	0.00
MicroVal 2D Mesh	PP	T	0.182 ± 0.028	0 (t = 250 s)	100.00	3.64	0.00
Parietex Composite	PET/collagen	L	0.064 ± 0.008	0.041	35.93	1.28	0.82
Parietex Composite	PET/collagen	T	0.039 ± 0.013	0 (t = 151 s)	100.00	0.78	0.00
Surgimesh	PP	L	0.111 ± 0.050	0.059	46.85	2.22	1.18
Surgimesh	PP	T	0.168 ± 0.040	0.082	51.19	3.36	1.64
Surgipro	PP	L	0.144 ± 0.029	0.058	59.72	2.88	1.16
Surgipro	PP	T	0.321 ± 0.120	0.128	60.12	6.42	2.56
TecnoMesh	PP	L	0.187 ± 0.060	0.080	57.22	3.74	1.60
TecnoMesh	PP	T	0.511 ± 0.040	0.218	57.33	10.22	4.36
Optilene	PP	L	0.187 ± 0.149	0 (t = 180 s)	100.00	3.74	0.00
Optilene	PP	T	1.028 ± 1.270	0.542	47.28	20.56	10.84
Parietex LW	PET	L	0.429 ± 0.040	0.306	28.67	8.58	6.12
Parietex LW	PET	T	0.245 ± 0.020	0.184	24.90	4.90	3.68
Ultrapro	PP/PGC-25	L	0.141 ± 0.016	0 (t = 300 s)	100.00	2.82	0.00
Ultrapro	PP/PGC-25	T	0.154 ± 0.003	0.044	71.43	3.08	0.88
Vypro II	PP/PG 910	L	0.510 ± 0.240	0.412	19.22	10.20	8.24
Vypro II	PP/PG 910	T	0.098 ± 0.030	0.078	20.41	1.96	1.56

Note: Orthogonal directions: Longitudinal (along the loop columns; L direction) and transverse (across the loop columns; T direction). Initial stress (T_0_), equilibrium stress (T_eq_), modulus at initial strain (E_0_), modulus at equilibrium state (E_eq_), the level of orthotropy of meshes, and stress reduction parameter ΔT.

## Data Availability

No new data were created or analyzed in this study. Data sharing is not applicable to this article.
